# Drug dosing in hospitalized obese patients with COVID-19

**DOI:** 10.1186/s13054-022-03941-1

**Published:** 2022-03-14

**Authors:** Jeffrey F. Barletta, Brian L. Erstad

**Affiliations:** 1 Department of Pharmacy Practice, College of Pharmacy, Midwestern University, 19555 N 59th Avenue, Glendale, AZ 85038 USA; 2 Department of Pharmacy Practice and Science, College of Pharmacy, University of Arizona, 1295 N Martin Ave, PO Box 210202, Tucson, AZ 85721 USA

**Keywords:** COVID-19, Obesity, Dosing, Dexamethasone, Baricitinib, Tofacitinib, Tocilizumab, Sarilumab, Remdesivir

## Abstract

Obesity is highly prevalent in hospitalized patients admitted with COVID-19. Evidence based guidelines are available for COVID-19-related therapies but dosing information specific to patients with obesity is lacking. Failure to account for the pharmacokinetic alterations that exist in this population can lead to underdosing, and treatment failure, or overdosing, resulting in an adverse effect. The objective of this manuscript is to provide clinicians with guidance for making dosing decisions for medications used in the treatment of patients with COVID-19. A detailed literature search was conducted for medications listed in evidence-based guidelines from the National Institutes of Health with an emphasis on pharmacokinetics, dosing and obesity. Retrieved manuscripts were evaluated and the following prioritization strategy was used to form the decision framework for recommendations: clinical outcome data > pharmacokinetic studies > adverse effects > physicochemical properties. Most randomized controlled studies included a substantial number of patients who were obese but few had large numbers of patients more extreme forms of obesity. Pharmacokinetic data have described alterations with volume of distribution and clearance but this variability does not appear to warrant dosing modifications. Future studies should provide more information on size descriptors and stratification of data according to obesity and body habitus.

**Graphic Abstract:**

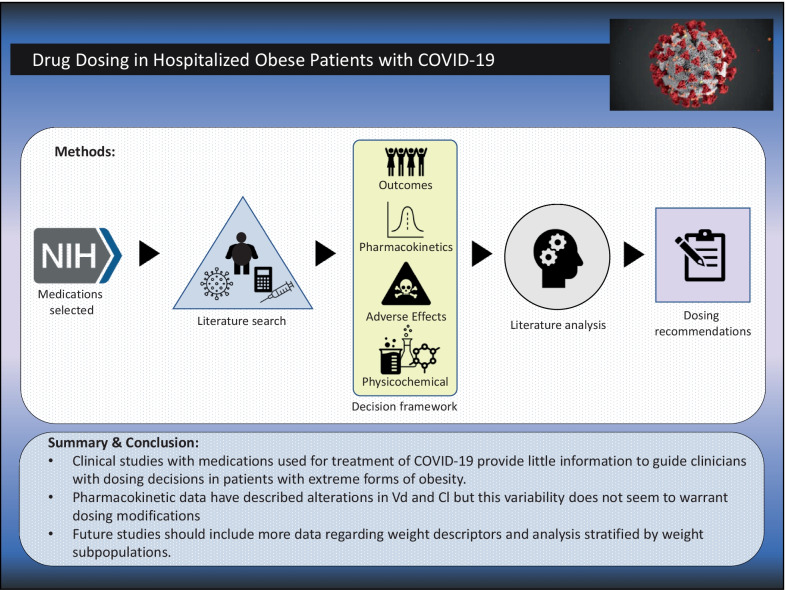

## Background

Obesity is widely recognized as a risk factor for severe COVID-19 infection with a greater risk for mortality compared to non-obese individuals. In fact, with each 1 kg/m^2^ increase in body mass index, the risk for severe infection has been reported to increase by 9% and mortality by 6% [1]. This contradicts the obesity paradox that has been described in other critically ill populations [2]. Reasons for this are multifactorial and include metabolic dysfunction, immune impairments, adipose inflammation and the presence of other related comorbidities (e.g., hypertension, diabetes, hyperlipidemia) [3].

The increasing prevalence of patients with COVID-19 and extreme obesity (i.e., BMI ≥ 40 kg/m^2^) has presented numerous challenges, one of which being medication dosing. Obesity can have a profound effect on drug pharmacokinetics particularly volume of distribution and clearance. Volume of distribution is the most important parameter when a single dose of a medication is given (e.g., a loading dose) while clearance becomes more influential with repeated or maintenance dosing. In general, drugs with a smaller volume of distribution are hydrophilic, with limited distribution into adipose tissue. Drugs with a larger volume of distribution tend to be more lipophilic, although exceptions do exist. Obesity is also associated with an increase in kidney mass but the effect on clearance is complex. Obesity, by itself is a risk factor for both chronic kidney disease and acute kidney injury and assessment of kidney function can be influenced by both assessment and indexing strategies [4–6]. Increases in clearance have been reported which could be due to the increase in renal blood flow [7]. For most medications, though the increase in clearance is not proportional to the increase in total body weight. Studies describing clearance have produced mixed results making the impact of obesity much more difficult to predict.

The lack of obesity-specific dosing recommendations is concerning because, drug doses typically originate from pharmacokinetic studies conducted in healthy individuals with normal body habitus. They do not account for the pharmacokinetic variability noted in patients with obesity, which could lead to concentrations outside the therapeutic range, treatment failure and/or an adverse event. For example, with some medications, distribution and clearance will increase substantially with increases in weight. With the use of fixed or non-weight-based dosing strategies, subtherapeutic concentrations are possible. Conversely, with other medications, there are no substantial changes in distribution and clearance with increasing weight. In the latter example, the use of weight-based dosing strategies according to total body weight could lead to supratherapeutic concentrations. Another challenge is the inability of existing weight metrics to distinguish fat-mass from fat-free mass. For example, take three hypothetical patients, all of which are the same age, sex and height, and weigh 100 kg. One patient is a body builder with extensive muscle mass, the second has increased fluid retention, while the third has increased fat-mass secondary to obesity. Despite being the same weight, there are clear differences in body composition that would affect drug distribution and clearance. Drug physicochemical properties are also relevant and can influence distribution. Physicochemical properties include physical and chemical properties of a drug that influence drug pharmacokinetics, such as lipophilicity, which is assessed using a partition co-efficient (log P). A log P of zero indicates the drug is equally partitioned between lipid and aqueous phases with higher positive values reflecting a greater degree of lipophilicity. While a complete review of pharmacokinetic considerations with obese patients is beyond the scope of this manuscript, the reader is referred elsewhere [8].

Crafting evidence-based dosing regiments for patients with COVID-19 is complicated because most of the therapies used in for management are off-label and in many cases extrapolated from studies in other disease states. Recommendations for COVID-19 drug therapy are available from several organizations, but none describes strategies for dosing that are specific for obese patients. With up to 50% of patients admitted to an ICU having a BMI > 30 kg/m^2^, there is a need for clear guidance with crafting dosing regimens in this challenging population [9].

This narrative review will describe the literature pertinent to drug dosing in obesity as it applies to COVID-19 related therapies with a focus on medications recommended by the National Institutes of Health (NIH) for hospitalized adults [10]. The purpose is to provide a framework to help clinicians make dosing decisions in this population despite the lack of high-quality outcome data. While there are many other adjunctive medications frequently used in these patients (i.e., sedatives, analgesics, neuromuscular blockers, anticoagulants), these will not be discussed, and the reader is referred to other thorough reviews [11–13].

The medications chosen for inclusion in this review were those listed in guidelines from the NIH for therapeutic management in hospitalized adults: dexamethasone, baricitinib, tofacitinib, tocilizumab, sarilumab, and remdesivir. For each medication, a detailed literature search was performed using PubMed from inception to November, 2021 and the search terms from the following three categories: (1) obesity: "Obesity"[Mesh] OR "Overweight"[Mesh] OR "body composition"[MeSH Terms] OR "extreme obesity" OR "body weight change*" OR "body size" OR "body fat" OR "body fatness" (2) pharmacokinetics and dosing: "Drug Monitoring"[Mesh] OR "Dose–Response Relationship, Drug"[Mesh] OR "pharmacokinetic" OR "pharmacokinetic considerations " OR "drug dosing" OR "drug dose" OR "therapeutic drug monitoring" OR "drug monitoring", (3) COVID-19: “COVID 19”[Mesh] OR “SARS-CoV-2”[Mesh] OR “2019-nCoV” OR “coronavirus disease”. The results from the primary literature search were reviewed along with bibliographies of both retrieved articles and the NIH guidelines to capture any articles that may have been missed by the primary literature search. Animal studies were not included. The emphasis was on extreme obesity (BMI ≥ 40 kg/m^2^), since these patients are often not well represented in either clinical or pharmacokinetic studies that led to the dosages listed in the product label. Medication-specific suggestions utilized a framework for decision making based the following prioritization strategy: clinical outcome data > pharmacokinetic studies > adverse effect profiles > physicochemical properties. DrugBank, a comprehensive, online database of detailed drug chemical, pharmacological and pharmaceutical data, was used for physicochemical properties (e.g., log P) unless otherwise referenced [14]. Because of the heterogeneity of outcome measures and the expected lack of information for many of the medications included, no quantitative analysis (meta-analysis) was performed.

## Main text

### Dexamethasone

Dexamethasone is a long-acting corticosteroid that has been widely used in patients with severe COVID-19 infection (i.e., those requiring supplemental oxygen). The benefit of corticosteroids has been described in several randomized controlled trials, most notably being the RECOVERY trial [15]. This trial randomized 6425 patients in a 2:1 ratio to receive either dexamethasone 6 mg or placebo. Dexamethasone was associated with a significant improvement in mortality in patients requiring mechanical ventilation or supplemental oxygen. Patient weight however was not reported therefore it is unknown if patients with obesity were well represented in this study cohort. Another smaller study evaluated dexamethasone and similar to the RECOVERY trial, a benefit in outcome was observed [16]. Patient weights were not reported. Recently, dexamethasone dose was evaluated in a randomized controlled trial comparing 6 mg with 12 mg [17]. In this trial, the median number of days alive without life support was 22 versus 20.5 days (adjusted mean difference = 1.3 (0–2.6) days. The median and upper quartile for weight in this trial was 80 and 96 kg, respectively, which likely indicates a small proportion of patients with extreme obesity were included.

Dexamethasone, like other corticosteroids, is a lipophilic compound with a log P of 1.83. Only one study has reported dexamethasone pharmacokinetics in 8 obese patients and 6 healthy controls [18]. No difference was noted in maximum plasma concentration. Similarly, pharmacokinetic studies with other corticosteroids have suggested dosing adjustments secondary to weight are unnecessary. Standard doses of dexamethasone similar to those used in non-obese patients should be utilized in obese patients. A 12 mg dose could be justified, not necessarily based on weight, but on possible benefit that has been observed.

### Janus kinase inhibitors

#### Baricitinib

Baricitinib is a janus kinase inhibitor that can modulate the immunological and inflammatory response encountered following COVID-19 infection. There are two randomized controlled trials evaluating baricitinib in patients with severe COVID-19. In the first, patients were randomized to receive either baricitinib 4 mg daily plus remdesivir or remdesivir alone; glucocorticoid use was restricted [19]. The median BMI was 31 kg/m^2^ with an interquartile range of 9 kg/m^2^ indicating a large proportion of patients were obese but a limited number had more extreme forms of obesity. Nonetheless, the combination of baricitinib and remdesivir was superior to remdesivir alone in time to recovery and improvement in clinical status. A second study randomized patients to receive baricitinib 4 mg daily or placebo in combination with standard of care (including corticosteroids) [20]. The mean BMI in this study was 30 ± 6 kg/m^2^ with 33% being categorized as obese. Baricitinib was associated with no difference in disease progression but a significant reduction in mortality was observed.

Baricitinib has a large volume of distribution (76 L) with a log P of 1.08. Dosing in adult patients with COVID-19 is non-weight based adjusted to estimated glomerular filtration rate. The package insert states body weight does not have a clinically relevant effect on area under the curve (AUC) or maximum concentration but this is in reference to a 100 kg patient compared to a comparator of 70 kg [21]. One study in pediatric patients showed a significant association between weight and volume of distribution [22]. While clearance did increase with each weigh category (< 20 kg, 20–40 kg, > 40 kg), that increase did not appear to be proportionate (8.4 L/h, 9.2 L/h and 13.9 L/h, respectively). Note, these data may not be generalizable to adult patients with more extreme forms of obesity. Nevertheless, the effect of weight-based dosing adjustments remain unknown; standard doses are therefore suggested. Further research is needed.

#### Tofacitinib

Tofacitinib is recommended as an alternative to baricitinib when baricitinib is not available or not feasible to use. One randomized controlled trial compared tofacitinib with placebo and a significant reduction in death or respiratory failure at day 28 was observed [23]. The median (IQR) BMI was 29.7 (26.7–32.9) kg/m^2^ indicating about half the study cohort were obese but a small number had more extreme forms of obesity.

Similar to baricitinib, tofacitinib has a large volume of distribution (87 L) with a log P of 1.58. One pharmacokinetic study in patients with psoriatic arthritis concluded tofacitinib doses do not require modification based on body weight [24]. The average weight in this study was 85 kg ranging from 38 to 160 kg. Similarly, a second pharmacokinetic study reported systemic exposures for patients in the 90th percentile for body weight (117 kg) were similar to that observed in patients in the 50th percentile (86 kg) [25]. Standard doses should be utilized.

### IL-6 inhibitors

#### Tocilizumab

Tocilizumab is a monoclonal antibody that inhibits binding of IL-6 to membrane and soluble IL-6 receptors to modify immune and inflammatory responses. Tocilizumab is primarily used in inflammatory disorders like rheumatoid arthritis and cytokine release syndrome following chimeric antigen receptor T-lymphocyte (CAR-T) cell therapy but studies in COVID-19 have demonstrated a beneficial effect on patient outcomes [26]. Several prospective, randomized controlled trials exist evaluating tocilizumab in patients with varying degrees of disease severity (Table 1) [27–37].

**Table 1:** Characteristics of weight in subjects included in randomized controlled trials evaluating tocilizumab

Study	*N*	Dose	Weight descriptor for tocilizumab arm	Main results
BACC Bay Stone [27]	243	8 mg/kg, max 800 mg	BMI = 29.9 (26–34.2) kg/m^2^	No difference in mechanical ventilation or death
CORIMUNO-19 Hermine [28]	131	8 mg/kg, option for additional 400 mg dose at 72 h	Weight = 80 (70–90) kg BMI = 27.9 (23.3–30.8) kg/m^2^	No improvement in need for mechanical ventilation on day 4 or survival free from mechanical ventilation
COVACTA Rosas [29]	438	8 mg/kg, max 800 mg, option to repeat dose in 8–24 h	Weight = 89 ± 24 kg	No difference in clinical status
COVINTOC Soin [30]	180	6 mg/kg, max 480 mg, option to repeat dose in 12 h	Not reported	No improvement in progression of disease
EMPACTA Salama [31]	340	8 mg/kg, max 800 mg, option to repeat dose in 8–24 h	Weight = 90 ± 24 kg BMI = 32 ± 7.9 kg/m^2^	Reduction in progression to the composite of mechanical ventilation or death
RCT-TCZ-COVID-19 Salvarani [32]	126	8 mg/kg, max 800 mg followed by 2nd dose after 12 h	BMI ≥ 30 kg/m^2^ = 28%	No difference in clinical worsening
RECOVERY [33]	1350	800 mg if > 90 kg, 600 mg if > 65 and ≤ 90 kg, 400 mg if > 40 and ≤ 65 kg, 8 mg/kg if ≤ 40 kg, option to repeat dose in 12–24 h	Not reported	Improvement in 28-day survival
REMAP-CAP, 2021 [34]	865	8 mg/kg, max 800 mg, option to repeat dose in 12–24 h	BMI = 30.5 (26.9–34.9) kg/m^2^	Improvement in respiratory and cardiovascular organ support-free days
REMDACTA Rosas [35]	649	8 mg/kg, max 800 mg, option to repeat dose in 8–24 h	Weight = 94 ± 27 kg	No difference in time to discharge
TOCIBRAS Veiga [36]	129	8 mg/kg, max 800 mg	Obesity = 23%	No improvement in clinical status at day 15
Wang [37]	65	400 mg, option to repeat dose at 24 h	Not reported	No difference in cure rate

Weight descriptors reported as either mean ± standard deviation, median (interquartile range)

In most trials, the dose of tocilizumab was 8 mg/kg with a maximum dose of 800 mg. A weight descriptor was provided in a few of the studies, whereby obese patients (i.e., BMI ≥ 30 kg/m^2^) made up a substantial proportion of patients included in the study cohort. The inclusion of patients with more extreme forms of obesity (i.e., BMI ≥ 40 kg/m^2^) however was limited.

Tocilizumab has a small volume of distribution (6.4 L) and like other monoclonal antibodies is hydrophilic. Tocilizumab is eliminated through two mechanisms. The first involves engagement on both soluble and membrane bound receptor targets followed by drug degradation; this process is non-linear and can be influenced by patient-specific factors such as severity of disease [38]. The second is through catabolism via the reticuloendothelial system, which is linear. At low concentrations, non-linear clearance is predominant but at higher concentrations this mechanism becomes saturated and linear clearance becomes the principal pathway. Pharmacokinetic studies have shown linear clearance to be correlated with weight but not in a proportional manner [39]. This could lead to over-exposure with weight-based dosing (and no dose cap) in patients with obesity. The concept of fixed dosing regimens has been proposed, however these models were not inclusive of patients with extreme obesity [40, 41]. One simulation-study reported predicted tocilizumab concentrations following weight-based (8 mg/kg) and various fixed dosing regimens (i.e., 400 mg, 600 mg, 800 mg) across a range of patient weights (41–160 kg) [38]. A reference range for tocilizumab exposure was extrapolated from data obtained from the REMAP-CAP study [34]. An 800 mg dose, the dosing cap utilized in most studies, did achieve target AUC and minimum concentration all weight stratums including the highest (126–160 kg). Lower fixed doses (400 mg and 600 mg) were insufficient in this cohort. Furthermore, in each of the simulated models, an 8 mg/kg weight-based regimen was associated with increasing exposure with increasing weight. Based on the available data, the 800 mg dose cap used in clinical trials (which mimics a fixed dosing strategy when weight exceeds 100 kg) appears appropriate, at least in patients up to 160 kg. Redosing was permitted in most of the clinical trials, which could be considered if clinical improvement is not observed. Further research is necessary to evaluate if higher doses are required in patients with weights exceeding 160 kg.

#### Sarilumab

Sarilumab is an IL-6 receptor antagonist that is recommended as an alternative to tocilizumab in situations where tocilizumab is not available. One randomized controlled trial revealed sarilumab was associated with improvements in the composite of mortality and the need for organ support versus control with similar effectiveness compared to tocilizumab [42]. In this trial, the median (IQR) BMI for patients receiving sarilumab was 31.2 (27.7–36.3) kg/m^2^. Other studies have not demonstrated benefit with sarilumab [43, 44]. In these studies, the percentage of patients with a BMI in excess of 30 kg/m^2^ was 42% and 22%, respectively.

Sarilumab is a hydrophilic compound with a small volume of distribution (7.3 L). The recommended dose for COVID-19 is a single, fixed dose of 400 mg. One pharmacokinetic study, obtained from patients with rheumatoid arthritis and normal body habitus, reported about a 20% reduction in AUC with a weight change from 71 to 83 kg [45]. A second study described sarilumab exposure–response relationships in patients with rheumatoid arthritis [46]. In this study, body weight was not associated with a significant change in the pharmacokinetic/pharmacodynamic model. Recommendations listed in the product label state although body weight influences sarilumab pharmacokinetics, no dose adjustments are required [47].

### Antiviral therapy

#### Remdesivir

Remdesivir inhibits viral replication by inhibiting SARS-CoV-2 RNA-dependent RNA polymerase. The approved product labeling for remdesivir restricts administration by IV infusion to patients weighing at least 40 kg in addition to age and renal function limitations (i.e. at least 12 years with an estimated glomerular filtration rate of at least 30 mL/min [48]. Remdesivir is a prodrug that undergoes metabolism to its active form active form GS-441524. While the latter metabolite is filtered by the glomerulus, toxicity concerns of a renally-cleared excipient (betadex sulfobutyl ether sodium) in the remdesivir formulation accounts for the recommendation to avoid use of the drug in patients with an estimated glomerular filtration rate less than 30 mL/minute. The dosing of remdesivir is non-weight based (200 mg load then 100 mg once daily) and the product labeling has no information related to dosing in obesity. Four trials serve as the basis for the remdesivir recommendations in the NIH guidelines [49–52]. In the Adaptive COVID-19 Treatment Trial (ACTT-1), patients with severe COVID-19 were randomized to receive remdesivir or placebo for a maximum of 10 days [49]. Obesity was an admitting condition in 45% of the 541 patients receiving remdesivir with a median (range) body mass index (BMI) of 29.2 kg/m^2^ (14.4–69.5 kg/m^2^). The mean (SD) and median (range) weight of patients receiving remdesivir was 89.5 (24.77) kg and 85.3 (41.7–238.1) kg, respectively. In a second trial, patients with moderate COVID-19 were randomized to receive a 5 (*n* = 199) or 10 (*n* = 197) day course of remdesivir or usual care (*n* = 200) [50]. The median (IQR) BMIs of the patients receiving remdesivir were 27 (24–30) kg/m^2^ and 28 (25–32) kg/m^2^ for patients receiving the 5 and 10 day courses, respectively. In the other two trials (the multicenter randomized World Health Organization Solidarity Trial and a multicenter cohort investigation conducted in Denmark) serving as the basis for the NIH recommendations concerning remdesivir, there was no reporting of size descriptors in the resultant publications [51, 52].

While the prodrug remdesivir is relatively lipophilic with a log P of 1.6, its active metabolite is more than 100 times as water soluble with a log P of − 1.09 [53]. Modeling data suggests that the lipophilic nature of the parent compound has potential pharmacokinetic implications in obese compared to normal weight patients, such as an increase in volume of distribution and AUC time curve including greater uptake into the central nervous system with reduced systemic clearance. While additional studies are warranted, the substantial number of obese subjects in the ACTT-1 trial at least lends support to the currently recommended fixed-dose regimen for patients with less extreme forms of obesity [49].

## Summary and conclusion

The studies serving as the basis for the drug recommendations in the NIH guidelines for hospitalized patients with COVID-19 provide little guidance for clinicians on the applicability of available dosing information for patients with more extreme forms of obesity. This problem is not unique to drug therapy for COVID-19 management, since drug dosing in obesity is not a required subsection of the section titled USE IN SPECIAL POPULATIONS in FDA approved labeling, a section which typically contains information on special populations such as pregnancy, lactation, pediatrics, and renal or hepatic impairment. In addition to improvements in product labeling, future investigations should attempt to enroll diverse populations that include patients of more extreme body habitus. Regardless of study size and diversity of enrollment, investigations should try to provide more details on size descriptors such as weight and BMI. In smaller studies such as pharmacokinetic evaluations, there should be a listing of patient-specific size descriptor data. In studies with larger sample sizes such as multicenter randomized controlled trials, the breakdown could be by categorical groupings in addition to mean (SD) and median (range). Given the possible extremes of BMIs, the BMI categorization should not only include the usual normal, overweight, and obese categories, but also subcategorization of BMI recordings such as groupings for every 10 kg/m^2^ above 40 kg/m^2^.

## Data Availability

The data supporting the conclusions of this article were obtained from previously published peer-reviewed articles and retrieved via a literature search and outlined in the bibliography. There were no original data used to formulate the conclusion.

## Abbreviations

ICU: Intensive care unit
BMI: Body mass index
NIH: National Institutes of Health
AUC: Area under the curve
